# Contribution of Five Functional Loci of Dopamine Metabolism-Related Genes to Parkinson’s Disease and Multiple System Atrophy in a Chinese Population

**DOI:** 10.3389/fnins.2020.00889

**Published:** 2020-09-03

**Authors:** Yongping Chen, Ruwei Ou, Lingyu Zhang, Xiaojing Gu, Xiaoqin Yuan, Qian-qian Wei, Bei Cao, Bi Zhao, Ying Wu, Huifang Shang

**Affiliations:** ^1^Department of Neurology, Rare Disease Center, West China Hospital, Sichuan University, Chengdu, China; ^2^National Clinical Research Center for Geriatric, West China Hospital, Sichuan University, Chengdu, China

**Keywords:** Parkinson’s disease, multiple system atrophy, genetic analysis, case-control association study, dopamine metabolism genes

## Abstract

**Background:** Impaired dopamine metabolism is associated with Parkinson’s disease (PD). Considering the overlap in the clinical and pathological characteristics between PD and multiple system atrophy (MSA), we investigated the effect of five potential functional polymorphisms in dopamine metabolism-related genes on disease susceptibility, phenotypes, and responses to dopamine in a large sample of PD and MSA patients.

**Methods:** A total of 1506 PD patients, 496 MSA patients, and 894 healthy controls were included in this study. Five variants (rs6356 in *TH*, rs921451 in *DDC*, rs4680 in *COMT*, rs1799836 in *MAOB*, and rs1611115 in *DBH*) were genotyped in all cases using Sequenom iPLEX Assay technology.

**Results:** After adjusting for gender and age at onset, except for *DDC* rs921451, which was associated with an increased risk of MSA (*p* = 0.001, OR = 1.21), no significant differences were found in genotype distribution or minor allele frequencies for the other four variants between PD and MSA patients and healthy controls. In the subgroup analysis, *DDC* rs921451 was associated with an increased risk for late-onset PD as well as for PD onset in males (*p* = 0.002 [OR = 1.13] *p* = 0.003 [OR = 1.15], respectively). In addition, patients harboring the risk allele *DDC* rs921451 required lower levodopa equivalent daily doses of dopaminergic medication than those without the risk allele (52.00 ± 21.31 mg/day, *p* = 0.015).

**Conclusion:** None of the five candidate functional variants is a major determinant of the risk for PD or MSA. The modified PD phenotypes associated with these variants requires further confirmation.

## Introduction

Parkinson’s disease (PD) is the second most common neurodegenerative disorder after Alzheimer’s disease and is characterized by both motor and non-motor symptoms. The progressive degeneration of dopaminergic neurons is the most important pathological hallmark of PD (Dauer and Przedborski, 2003). Although the exact pathogenesis of neurodegeneration is largely unknown, the impaired activity of dopamine metabolism-related enzymes is known to be associated with PD physiopathology and phenotypes (Devos et al., 2014; Redensek et al., 2019).

Neurotransmitter metabolism involves both anabolic and catabolic processes. Dopamine anabolism involves two rate-limiting enzymes, tyrosine hydroxylase encoded by the *TH* gene and aromatic L-amino acid decarboxylase encoded by the *DDC* gene, and dopamine catabolism is associated with the activities of catechol-*O*-methyltransferase (encoded by the *COMT* gene), monoamine oxidase B (encoded by the *MAOB* gene), and dopamine beta-hydroxylase (encoded by the *DBH* gene). Studies have reported that the functional polymorphisms rs6356 in *TH*, rs921451 in *DDC*, rs4680 in *COMT*, rs1799836 in *MAOB*, and rs1611115 in *DBH* are associated with the activities of their respective enzymes and influence PD susceptibility and/or its phenotypes (Kurth et al., 1993; Healy et al., 2004).

Rs6356, located in exon 2 of *TH*, represents a V81M substitution in the regulatory domain of the tetrameric enzyme, which seems to have an inhibitory effect on enzyme function (Kumer and Vrana, 1996). A recent study reports that rs6356 plays a protective role in late-onset PD, suggesting that the activity of the enzyme is important in aging (Yan et al., 2018). Rs921451, located in the promoter of the *DDC* gene, may cause a reduction in DDC enzyme expression (Devos et al., 2014). DDC is proposed to be involved in the development of PD or the drug response of PD patients to levodopa, which is consistent with the finding that carriers of the risk allele *DDC* rs921451 have a less intense motor response (Devos et al., 2014). The risk allele “A” of rs4680 (V158M) in *COMT* has been associated with low activity of soluble COMT (Syvanen et al., 1997) and may be linked to an increased risk of developing PD. One study identifies that the risk allele “G” of the *MAOB* rs1799836 polymorphism is associated with lower MAOB activity in the brain (Balciuniene et al., 2002). Moreover, based on a meta-analysis, the “G” allele is also suggested to be involved in a genetic predisposition for PD (Sun et al., 2014). However, this observation is controversial because there was no sex composition ratio for patients or controls, and the *MAOB* gene is located on the sex chromosome. In addition, the minor “T” allele at rs1611115, located within the promoter of the *DBH* gene, is linked with low serum DBH activity and protects against PD (Healy et al., 2004), indicating that it is a marker for the causal variant.

Multiple system atrophy (MSA), another synucleinopathy, is a fatal adult-onset neurodegenerative disorder that shares some clinical manifestations and pathophysiology with PD (Wong and Krainc, 2017). Dopamine replacement is a primary therapeutic strategy for MSA because no curative treatment is yet available. Nevertheless, although approximately 30% of patients have an initial positive response to dopamine replacement, sustained response rates remain disappointing (Wenning et al., 1994). Research on dopamine metabolism is necessary because the tolerance level for dopamine replacement is still unknown.

The disease risk associated with these polymorphisms is largely unknown, especially in the Chinese population. Differences in sample size, race, and regional heterogeneity as well as the inclusion or not of subgroups have contributed to the identification of inconsistent or contradictory associations. In addition, response to dopamine preparation as well as non-motor symptoms, such as anxiety, depression, and cognitive function, should also be investigated owing to the large number of dopamine targets present in the central and peripheral nervous systems. Therefore, in the current study, we conduct a comparative association analysis of the polygenic effects on disease susceptibility, motor or non-motor symptoms, and response to dopamine in a large sample of PD and MSA patients.

## Materials and Methods

### Subjects

All the patients in this study were registered at the department of neurology at the West China Hospital of Sichuan University from May 2006 to December 2017. Two independent sets of patients were studied. The first group, comprising PD patients, was diagnosed according to the UK Parkinson’s Disease Society Brain Bank Clinical Diagnostic Criteria for PD (Hughes et al., 1992). The second group, consisting of probable MSA patients, met the current consensus criteria for MSA (Gilman et al., 2008); possible MSA patients were excluded from the study. The diagnoses of PD and MSA were made by movement-disorder specialists. Detailed clinical variables were recorded: sex, age, education level, age at onset, and initial symptoms. The Unified PD Rating Scale (UPDRS) part III score, the Unified MSA Rating Scale (UMSARS), or the modified Hoehn and Yahr (H&Y) staging was used to evaluate motor disability. As described in our previous studies (Guo et al., 2014a,b), depression was indicated if the Hamilton Depression Rating Scale (HDRS, 24 items) score was > 20 (Hamilton, 1967), and anxiety was suggested if the Hamilton Anxiety Rating Scale (HARS, 14 items) score was > 14 (Clark and Donovan, 1994). Meanwhile, cognitive function was assessed using the Montreal Cognitive Assessment (MoCA) (Cecchini et al., 2019), and cognition impairment was suggested if the MoCA score was < 26 (Nasreddine et al., 2005). The levodopa equivalent daily dose (LEDD) for PD was calculated by the commonly used formula given by Tomlinson et al. (2010). A group of unrelated, age- and gender-matched Chinese healthy controls (HCs) was also recruited as the control group. Neurologists confirmed that none of the HCs had a neurological disorder. Written informed consent was obtained from all the subjects. The study was approved by the ethics committee of West China Hospital, Sichuan University.

### Selection of Candidate Variants and Genetic Analysis

Genomic DNA was collected from peripheral blood leukocytes via standard phenol–chloroform procedures. In the current study, all the subjects were genotyped for five potential functional polymorphisms, namely rs6356 in *TH*, rs921451 in *DDC*, rs4680 in *COMT*, rs1799836 in *MAOB*, and rs1611115 in *DBH*, using Sequenom iPLEX assay technology (Sequenom, San Diego, CA, United States) according to the manufacturer’s instructions as per our previous report (Chen et al., 2018). In addition, we have uploaded the SNP data to a publicly available repository (https://www.synapse.org/). The link to the SNP data is https://www.synapse.org/#!Synapse:syn22274616/tables/.

### Statistical Analysis

Each single nucleotide polymorphism (SNP) was tested for Hardy–Weinberg equilibrium in controls, and differences in genotype frequencies between patients and controls were determined using the chi-square test. Minor allele frequencies (MAFs) and odds ratios (ORs) with a 95% confidence interval (CI) were estimated to determine the function of each SNP. Differences in continuous variables were compared using a *t*-test. To investigate the association of candidate single factor polymorphisms with the risk of the development of PD or MSA, logistic regression models were used to adjust for the other covariates. In the subgroup analysis, the *p*-values and ORs were determined by logistic regression analysis after adjusting for gender, age at onset, disease duration, or education to minimize the effects of these variables. A two-tailed *p*-value ≤ 0.05 was considered statistically significant. The Bonferroni method was used to counteract the problem of multiple comparisons. All analyses were performed using SPSS version 19.0 (SPSS, Inc., Chicago, IL, United States). Statistical power was calculated using PS Power and Sample Size Calculations software (version 3.0.43).

## Results

### Demographic Information

A total of 2002 patients, including 1506 PD patients, 496 MSA patients, and 894 HCs, were examined in this study. The demographic and clinical characteristics, including age, gender, mean age at onset, UPDRS-III/UMSARS, H&Y stage, and LEDD, are shown in Table 1. No significant differences were found in gender distribution, mean age at onset, or age between PD or MSA patients and HCs (*p* = 0.458 and 0.356, respectively). The mean disease duration was longer for PD patients than for MSA patients (*p* < 0.001).

**TABLE 1:** Demographic and clinical characteristics of all the subjects.

Variants	PD	MSA	HCs
Cases	1506	496	894
Female (%)	711(47.21)	246(49.61)	457(53.82)
Early onset (≤50%)	410, 27.22%	105, 21.17%	-
Age of sample collection (years, Mean ± SD)	60.60 ± 11.44	59.42 ± 8.62	56.23 ± 11.05
Mean onset age (years, Mean ± SD)	57.10 ± 11.42	56.73 ± 8.88	-
Disease duration (years, Mean ± SD)	3.83 ± 3.76	2.77 ± 1.93	
UPDRS-III/UMSARS*	29.05 ± 15.66	39.07 ± 18.65	-
H&Y stage	2.27 ± 0.90	-	-
LEDD (mg/d)	452 ± 263	-	-
Initial symptoms			
Tremor: rigidity/bradykinesia	733:532	-	-
MSA-C: MSA-P	-	255: 241	-

*For motor disability, PD patients were assessed by UPDRS-III, MSA patients were assessed by UMSARS; LEDD, levodopa equivalent daily dose.

### Genetic Findings

The genotyping call rate was more than 99% for each variant, and the genotype distribution of all the candidate variants in the HCs did not significantly deviate from Hardy–Weinberg equilibrium (Table 2). No significant differences were found in genotype distribution and MAFs for rs6356 in *TH*, rs921451 in *DDC*, rs4680 in *COMT*, and rs1611115 in *DBH* between PD patients and controls (for *MAOB* rs1799836, the comparison of genotype distribution and MAFs between patients and controls were according to different gender groups owing to this gene being located on the X chromosome; Tables 2, 3). However, the minor “C” allele of *DDC* rs921451 increased the risk for MSA (*p* = 0.001, OR = 1.21, 95% CI: 1.12–1.30). As shown in Supplementary Table 1, after adjusting for the other covariates, *DDC* rs921451 was an independent risk factor for MSA (the ORs of the heterozygous genotype and minor homozygous genotype of *DDC* rs921451 relative to the major homozygous genotype were 1.35 and 1.60, respectively). However, this difference in allele distribution for *DDC* rs921451 was only found in males in the gender subgroup analysis (*p* = 0.003, OR = 1.15, 95% CI: 1.05–1.31). In haplotype analysis, “C-T” and “T-T” in *TH* rs6356 and *DDC* rs921451 decreased the risk for PD and MSA (Supplementary Table 2), respectively.

**TABLE 2:** Genotype and allele frequency distribution of four candidate SNPs in patients and controls.

SNPs	Call rate	EAF in Chinese	HWE	Allele	Allele frequencies				Genotype	Genotype frequencies and distribution			
					PD (%)	MSA (%)	HCs (%)	OR (95%CI)^		PD (%)	MSA (%)	HCs (%)	*p*-Value^
*DBH*													
rs1611115	99.76%	0.1947	0.24	T	500(16.7)	172(17.4)	298(16.7)	1.00[0.87–1.19] *	TT	43(2.9)	16(3.2)	20(2.2)	0.526*
				C	2502(83.3)	818(82.6)	1488(83.3)	1.06[0.86–1.26] ^#^	CT	414(27.6)	140(28.3)	258(28.9)	0.513^#^
									CC	1044(69.6)	339(68.5)	615(68.9)	
*COMT*													
rs4680	99.90%	0.2981	0.83	A	765(25.4)	251(25.3)	436(24.4)	1.06[0.93–1.18] *	AA	83(5.5)	32(6.5)	52(5.8)	0.426*
				G	2243(74.6)	741(74.7)	1350(75.6)	1.03[0.90–1.18]^#^	AG	599(39.8)	187(37.7)	332(37.2)	0.848^#^
									GG	822(54.7)	277(55.8)	509(57.0)	
*TH*													
rs6356	99.59%	0.1947^&^	0.38	C	509(17.0)	173(17.5)	306(17.2)	0.97[0.85–1.13] *	CC	52(3.5)	17(3.4)	30(3.4)	0.928*
				T	2493(83.0)	813(82.5)	1474(82.8)	1.03[0.84–1.20]^#^	CT	405(27.0)	139(28.2)	246(27.6)	0.951^#^
									TT	1044(69.6)	337(68.4)	614(69.0)	
*DDC*													
rs921451	99.97%	0.4784	0.29	C	1538(51.1)	541(54.6)	877(49.0)	1.05[0.94–1.15] *	CC	417(27.7)	148(29.9)	223(24.9)	0.324*
				T	1474(48.9)	449(45.4)	911(51.0)	**1.21[1.12–1.32]^#$^**	CT	704(46.7)	245(49.5)	431(48.2)	0.021^#^
									TT	385(25.6)	102(20.6)	240(26.8)	

EAF in Chinese: effect allele frequency in Han Chinese according to 1000 genomes database; HWE: Hardy–Weinberg equilibrium in healthy controls group;*comparison between PD and HCs; ^#^comparison between MSA and HCs; ^&^the minor allele in Chinese is the major allele in Caucasian; ^$^*p* = 0.001; ^adjusted sex and onset age; Chi-squared test, the significant level was corrected with the formula of α’ = α/8 = 0.00625(8 tests for each disease in the table, 4 SNPs × 2 tests/SNP) according to the Bonferroni method; bold indicated significant differences after Bonferroni correction. The underline values means the minor allele of SNPs.

**TABLE 3:** Association analysis of five candidate SNPs in patients and controls according to sex.

	Genotype/allele	Male					Female				
		PD (*n* = 795)	MSA (*n* = 250)	HCs (*n* = 437)	*p*-Value^&^	OR (95%CI)^&^	PD (*n* = 711)	MSA (*n* = 246)	HCs (*n* = 457)	*p*-Value^&^	OR (95%CI)^&^
*MAOB*											
Rs1799836	CC	-	-	-			27(3.8)	9(3.7)	15(3.3)	0.606*	-
	CT	-	-	-			229(32.2)	83(33.7)	137(30.0)	0.534^#^	-
	TT	-	-	-			455(64.0)	154(62.6)	305(66.7)		
	C	146(18.4)	37(14.8)	97(22.2)	0.110*	0.82[0.65–1.03]	283(19.9)	101(20.5)	167(18.3)	0.329*	1.10[0.93–1.31]
	T	649(81.6)	213(85.2)	340(77.8)	0.014^#^	0.63[0.43–0.92]	1139(80.1)	391(79.5)	747(81.7)	0.312^#^	1.11[0.91–1.39]
*DBH*											
Rs1611115	T	255(16.1)	87(17.4)	131(15.0)	0.456*	1.07[0.89–1.30]	245(17.3)	85(17.3)	167(18.3)	0.545*	0.94[0.80–1.14]
	C	1331(83.9)	413(82.6)	743(85.0)	0.241^#^	1.16[0.91–1.49]	1171(82.7)	405(82.7)	745(81.7)	0.656^#^	0.93[0.74–1.19]
*COMT*											
rs4680	A	429(27.0)	124(24.8)	206(23.6)	0.071*	1.14[0.99–1.32]	336(23.7)	127(25.8)	230(25.2)	0.420*	0.95[0.82–1.10]
	G	1159(73.0)	376(75.2)	666(76.4)	0.614^#^	1.05[0.87–1.27]	1084(76.3)	365(74.2)	684(74.8)	0.778^#^	1.04[0.86–1.25]
*TH*											
Rs6356	C	283(17.8)	95(19.2)	150(17.2)	0.670*	1.04[0.87–1.24]	226(16.0)	78(15.9)	156(17.2)	0.456*	0.94[0.75–1.10]
	T	1305(82.2)	401(80.8)	722(82.8)	0.356^#^	1.11[0.88–1.40]	1188(84.0)	412(84.1)	752(82.8)	0.534^#^	0.92[0.70–1.17]
*DDC*											
Rs921451	C	823(51.8)	270(54.2)	408(46.7)	0.018*	1.10[1.01–1.20]	715(50.3)	271(55.1)	469(51.3)	0.615*	0.99[0.92–1.08]
	T	767(48.2)	228(45.8)	466(53.3)	**0.003^#^**	**1.15[1.05–1.31]**	707(49.7)	221(44.9)	445(48.7)	0.187^#^	1.06[0.96–1.18]

*Comparison between PD and HCs; #comparison between MSA and HCs; ^&^Chi-squared test, the significant level was corrected with the formula of α’ = α/10 = 0.005(10 tests for each disease in the table, 5 SNPs × 2 tests/SNP) according to the Bonferroni method, and the *p*-value was adjusted age; bold indicated significant differences after Bonferroni correction.

Increased anabolism or decreased catabolism of dopamine seems to affect the age at onset of the progressive degeneration of substantia nigra (SN) dopamine neurons. To elucidate this mechanism, the MAFs for all five variants were compared between early- or late-onset patients and the age at onset of patients with minor alleles or those without minor alleles. The minor allele of *DDC* rs921451 increased the risk of late-onset PD (Table 4; *p* = 0.002, OR = 1.13, 95% CI: 1.05–1.23), which was partly reflected in older ages at onset (0.99 ± 0.68 years) in patients with the minor allele compared with those without the allele; however, this difference was not significant (Table 5). Interestingly, significant differences were identified when a polygenic analysis was conducted. In the dopamine catabolic pathway, the age at onset was 2.51 ± 1.10 years earlier in patients with the *DBH* rs1611115 and *MAOB* rs1799836 risk alleles than in those without the alleles (*p* = 0.025). Additionally, this difference reached 3.22 ± 1.57 years in patients with the risk allele of all three dopamine catabolic-related variants compared to those without their risk allele (*p* = 0.041) (Table 5).

**TABLE 4:** Analysis of the minor allele frequency distribution of five candidate SNPs in patients regarding clinical presentation in PD.

Variants	*DBH* rs1611115		*MAOB* rs1799836			*COMT* rs4680		*TH* rs6356		*DDC* rs921451	
	MAF (%)	*p-*Value	MAF *(%)	MAF ^#^(%)	*p*-Value	MAF (%)	*p*-Value	MAF (%)	*p*-Value	MAF (%)	*p*-Value
**Onset age**^$^											
Early-onset (*n* = 410)	132(16.18)	0.632	44(20.47)	86(22.28)	0.372*	220(26.83)	0.255	158(19.32)	0.031	383(46.71)	**0.002^*Y–*^**
Late-onset (*n* = 1091)	367(16.87)		101(17.75)	186(18.09)	0.078^#^	542(24.86)		349(16.05)		1146(52.52)	
**Depression^**											
Presence (*n* = 317)	116(18.35)	0.311	27(19.29)	65(18.68)	0.737*	140(22.08)	0.044	104(16.51)	0.813	311(49.05)	0.289
Absence (*n* = 1047)	348(16.67)		102(18.12)	187(19.52)	0.729^#^	547(26.15)		353(16.91)		1077(51.43)	
**Anxiety^**											
Presence (*n* = 260)	94(18.15)	0.458	18(15.52)	60(21.13)	0.411*	115(22.12)	0.078	86(16.67)	0.916	257(49.42)	0.446
Absence (*n* = 1104)	370(16.8)		110(18.77)	192(18.79)	0.356^#^	572(25.93)		371(16.85)		1131(51.22)	
**MOCA**^&^											
Presence (*n* = 847)	303(17.97)	0.039	68(16.96)	165(18.79)	0.279*	406(23.97)	0.025	265(15.72)	0.039	867(51.58)	0.815
Absence (*n* = 647)	196(15.17)		75(19.84)	107(20.11)	0.528^#^	355(27.47)		239(18.50)		657(50.77)	

*In male; ^#^in female; ^$^*p*-value adjusted sex; ^*p*-value adjusted sex, onset age and disease duration; ^&^*p*-value adjusted sex, onset age, disease duration and education; ^*Y–*^OR = 1.13, 95%CI: 1.05–1.23; Chi-squared test, the significant level was corrected with the formula of α’ = α/24 = 0.0021(24 tests in the table, 4 SNPs × 4 tests/SNP and one SNP × 8 tests) according to the Bonferroni method; bold indicated significant differences after Bonferroni correction.

**TABLE 5:** Comparisons of the average onset-age between PD patients with risk and non-risk alleles of the loci.

Single SNP or multi-locus combination	Non-risk allele carriers		Risk allele carriers^		MD ± SD		*p*-Value^#^	
	Mean onset age	LEDD (mg/d)	Mean onset age	LEDD (mg/d)	Mean onset age	LEDD (mg/d)	Onset age*	LEDD^&^
	(mean ± SD/n)	(mean ± SD/n)	(mean ± SD)	(mean ± SD/n)				
**Catabolic pathway**								
rs1611115	57.21 ± 11.50, 1034	443.00 ± 264.54, 710	56.91 ± 11.27, 456	474.56 ± 288.95, 308	0.29 ± 0.64	31.56 ± 18.57	0.639	0.078
rs1799836	57.39 ± 11.46, 1097	458.78 ± 275.97, 762	56.30 ± 11.27, 398	429.01 ± 260.01, 251	1.09 ± 0.67	29.77 ± 19.80	0.111	0.141
rs4680	56.88 ± 11.24, 816	464.36 ± 286.97, 556	57.36 ± 11.64, 678	436.64 ± 252.62, 463	0.48 ± 0.59	27.72 ± 17.12	0.418	0.111
rs1611115 + rs1799836	57.32 ± 11.38, 1373	452.63 ± 271.71, 943	54.80 ± 11.71, 117	447.74 ± 278.66, 78	2.51 ± 1.10	4.89 ± 32.08	0.025	0.875
rs1611115 + rs4680	57.10 ± 11.40, 1297	453.85 ± 276.26, 874	57.20 ± 11.60, 210	442.73 ± 246.69, 147	0.10 ± 0.85	11.13 ± 24.27	0.913	0.636
rs1799836 + rs4680	57.23 ± 11.39, 1320	453.75 ± 271.80, 908	56.07 ± 11.68, 174	440.14 ± 275.52, 113	1.16 ± 0.92	13.62 ± 27.15	0.221	0.618
rs1611115 + rs1799836 + rs4680	57.23 ± 11.34, 1437	454.74 ± 273.63, 983	54.01 ± 11.35, 55	387.89 ± 222.94, 38	3.22 ± 1.57	66.85 ± 44.96	0.041	0.123
**Anabolic pathway**								
rs6356	57.37 ± 11.15, 1036	451.75 ± 257.02, 709	56.46 ± 12.04, 454	451.79 ± 304.07, 308	0.91 ± 0.64	0.04 ± 18.57	0.165	0.995
rs921451	56.36 ± 11.24, 383	491.42 ± 303.85, 252	57.35 ± 11.47, 1112	439.42 ± 259.81, 769	0.99 ± 0.68	52.00 ± 21.31	0.153	0.011
rs6356 + rs921451	57.17 ± 11.18, 1142	453.03 ± 261.73, 779	56.83 ± 12.23, 348	449.74 ± 303.70, 242	0.34 ± 0.70	3.29 ± 20.04	0.639	0.865

SD, standard deviation; MD, mean difference; SE, standard error difference; ^risk allele, represent minor allele; ^#^Logistic regression models for the differences between males with the risk and non-risk genotypes; *adjusted sex; ^&^adjusted sex, onset age, and disease duration.

The effects of these variants on PD-associated non-motor symptoms, motor complications, and response to dopaminergic preparations were also analyzed. We found that *COMT* rs4680 could decrease the risk for the manifestation of depressive symptoms in PD and that the minor alleles of *COMT* rs4680 and *TH* rs6356 might protect against the loss of cognitive function in PD patients. *DBH* rs1611115 was associated with an increased risk for impaired cognition in PD. However, these differences were not significant after correcting for multiple comparisons (Table 4). In addition, patients with the *DDC* rs921451 risk allele tended to need lower LEDDs of dopaminergic medication than patients without the risk allele (52.00 ± 21.31 mg/day) (Table 5). However, no significant differences were found in genotype distribution or MAFs for all five variants between patients presenting or not with motor complications, such as motor fluctuation, dyskinesia, freezing gait, and festinating gait (Supplementary Table 3).

## Discussion

In the current study, we found that five functional variants of dopamine metabolism-related genes did not affect the risk of developing PD; however, the minor allele “C” of *DDC* rs921451 increased the susceptibility to MSA, especially in males. Surprisingly, *DDC* rs921451 tended to be associated with increased age at onset for PD, and *DBH* rs1611115, *MAOB* rs1799836, and *COMT* rs4680 showed the opposite trend. In addition, patients carrying the *DDC* rs921451 risk allele might need lower LEDDs of dopaminergic drugs.

*TH* encodes the rate-limiting enzyme (a monooxygenase) in dopamine biosynthesis. Mutations in both alleles of *TH* have been associated with severe Parkinsonism-related phenotypes (Furukawa et al., 2001). Rs6356, the best-characterized SNP in *TH*, is postulated to be a variant with functional consequences and, therefore, more likely to confer genetic risk. In our study, we found that the rs6356 risk allele may increase the risk of early-onset PD and lower the age at onset for PD compared with patients without the allele; however, this was inconsistent with the results of a previous study showing that rs6356 was associated with late-onset PD in a cohort from southern China (Yan et al., 2018). Methodologically, we analyzed the mean age at disease onset for patients harboring or not the risk allele, and it is more logical that the risk allele of this variant decreases onset age as this gene is involved in dopamine synthesis. However, whether rs6356 affects the age at PD onset requires further investigation with a larger sample size.

In this study, we found that *DDC* rs921451 was associated with disease susceptibility or disease phenotype in both PD and MSA. Rs921451 has been reported to reduce the expression or activity of DDC (Devos et al., 2014), an enzyme involved in dopamine and norepinephrine (NE) synthesis (Bell and Mann, 1990). Reduced DDC expression results in Parkinsonism symptoms, and reduced DDC activity may have a role in vasodilatation and, consequently, hypotension, which are the features of autonomic dysfunction in MSA (Lau et al., 2018). Hence, it is not surprising that *DDC* rs921451 was found to increase the risk for MSA in our study, and the findings were also consistent with reports that *DDC* rs921451 increases the risk for orthostatic hypotension in PD (Redensek et al., 2019). However, the association was found only in males with MSA or PD. Several studies (Solla et al., 2012, 2020) show that gender-related differences in motor symptoms, non-motor symptoms, or other phenotypes might be due to differences in genetic background. Estrogen influences the synthesis, release, and metabolism of dopamine and modulates dopamine receptor expression and function (Shulman, 2002); therefore, it may modify the phenotype or affect the risk of gender differences. In addition, the finding that PD patients with the “C” risk allele require less dopaminergic medication than those without the risk allele could be explained by the protective effect resulting from low enzyme activity; this would avoid levodopa degradation in the periphery and lead to more levodopa passing through the blood–brain barrier into the central nervous system (CNS).

The broader role of dopamine in the CNS suggests that other phenotypes could be affected in PD, such as pain (Lin et al., 2017), daytime sleepiness (Rissling et al., 2006), and apathy (Cramer et al., 2010; Hong et al., 2015; Masala et al., 2018), all of which are very frequent symptoms in Parkinson’s disease patients as reported in previous studies. Although PD patients harboring *COMT* rs4680 seemed to have a reduced risk of developing depression, the minor *COMT* rs4680 and *TH* rs6356 alleles exerted a protective effect on cognitive function in PD patients, and *DBH* rs1611115 tended to increase the risk for impaired cognition in PD in the subgroup analysis. However, these findings need further confirmation because these differences disappeared after correction for multiple comparisons. In addition, although previous studies have reported that patients carrying *DDC* rs921451 have a less intense motor response and patients harboring *MAOB* rs1799836 develop levodopa-induced dyskinesia more frequently (Devos et al., 2014; Sampaio et al., 2018), we did not find any differences in genotype distribution between patients presenting or not with motor fluctuation, dyskinesia, freezing gait, or festinating gait.

To explore whether a combination of polymorphisms in these genes would show a stronger association with PD than the individual SNPs, we further investigated the joint effects of these variants on PD. The “C-T” haplotype associated with the *TH* rs6356 and *DDC* rs921451 risk alleles has a marginal level of association with a reduced risk for PD. Similarly, the “T-T” haplotype decreases the risk for MSA. In addition, although no significant effects of individual SNPs on the age at onset were identified, the age at onset of patients harboring the rs1611115 and rs1799836 risk alleles is approximately 2.5 years later than that of patients without these two risk allele variants together, and this difference reached 3.2 years if the patients carried all three risk allele variants of the catabolic-related genes. These risk alleles of individual catabolic-related genes that result in low enzyme activity are expected to have a compensatory role and delay the onset of PD symptoms (Ross et al., 2008), and it remains unclear why the joint effects of these variants should lead to an earlier age at onset in this study.

The strength of our study is that we include a relatively large and ethnically homogeneous cohort, and all the patients received regular, long-term follow-up. However, our study also has some limitations. To comprehensively investigate the relationship between dopamine metabolism and PD risk, other dopamine metabolism-related genes, such as those coding for dopamine transporters and receptors, should be analyzed. Second, in addition to gene–gene interactions, gene–environment effects should be examined. Third, considering the marginal levels of associations in some differences, replicate studies are warranted.

## Conclusion

Our results suggest that none of the five candidate functional variants of dopamine metabolism-related genes are major determinants of the risk for PD or MSA. The phenotypes of PD, including age at onset, depression, cognitive function, and dopamine preparation dosage, might be modified by these variants. Additional studies with a large sample size are required to reproduce our results.

## Data Availability Statement

The original contributions presented in the study are included in the article/Supplementary Material, further inquiries can be directed to the corresponding author.

## Ethics Statement

The studies involving human participants were reviewed and approved by West China Hospital, Sichuan University. The patients/participants provided their written informed consent to participate in this study.

## Author Contributions

YC: design, execution, data analysis, and writing. RO and LZ: execution and clinical data collection. XG: execution, blood sample collection, and DNA extraction. XY and QW: execution and clinical data collection. BC: execution, patient enrollment, and clinical data collection. BZ and YW: execution and patient enrollment. HS: conception, design, organization, review, and critique. All authors contributed to the article and approved the submitted version.

## Conflict of Interest

The authors declare that the research was conducted in the absence of any commercial or financial relationships that could be construed as a potential conflict of interest.
