# Supplementary material for: Contribution of Five Functional Loci of Dopamine Metabolism-Related Genes to Parkinson’s Disease and Multiple System Atrophy in a Chinese Population
Source: Front Neurosci. 2020 Sep 3;14:889. doi: 10.3389/fnins.2020.00889 (PMC7497786; doi:10.3389/fnins.2020.00889)
Supplement: Supplementary file 1 [file Data_Sheet_1.docx]

**Supplementary Table 1** single factor association with PD or MSA in the multivariable non-linear models

| Factors | PD | |  | MSA | |
| --- | --- | --- | --- | --- | --- |
|  | Multivariable p value | OR [95%CI] |  | Multivariable p value | OR [95%CI] |
| Sex | 0.10 | 0.87[0.73-1.03] |  | 0.69 | 0.96[0.72-1.20] |
| Age | 0.12 | 1.00[1.00-1.01] |  | 0.37 | 1.00[0.99-1.02] |
| *DBH* rs1611115: CT genotype | 0.67 | 0.96[0.80-1.16] |  | 0.84 | 0.98[0.76-1.25] |
| *DBH* rs1611115: CC genotype | 0.41 | 1.26[0.73-2.16] |  | 0.45 | 1.31[0.66-2.60] |
| *COMT* rs4680: AG genotype | 0.23 | 1.11[0.93-1.33] |  | 0.72 | 1.04[0.83-1.32] |
| *COMT* rs4680: AA genotype | 0.92 | 0.98[0.68-1.42] |  | 0.69 | 1.10[0.69-1.76] |
| *TH* rs6356: CT genotype | 0.70 | 0.96[0.80-1.16] |  | 0.79 | 1.03[0.81-1.33] |
| *TH* rs6356: CC genotype | 0.98 | 1.00[0.63-1.60] |  | 0.80 | 1.08[0.59-2.00] |
| *DDC* rs921451: CT genotype | 0.88 | 1.02[0.83-1.24] |  | **0.039** | **1.35[1.02-1.79]** |
| *DDC* rs921451: CC genotype | 0.28 | 1.14[0.90-1.43] |  | **0.004** | **1.60[1.17-2.19]** |
| *MAOB* rs1799836: C allele* | 0.73 | 0.97[0.80-1.17] |  | 0.46 | 0.91[0.70-1.17] |

*for male, there only are C allele or T allele, for female, there are CC, CT and TT genotype, therefore, T allele (including T allele in male and TT genotype) as reference, C allele (including C allele in male, C allele carriers (CT and CC genotypes)) as risk factor. For other four polymorphisms, the homozygous composed by major allele are the references genotype; Multivariable p value for each variable were adjusted for the other covariates; bold indicated significant differences p<0.05.

**Supplementary Table 2** Comparison of SNPs haplotype frequencies between PD patients and controls

| multi-locus combination | Case (freq) | Control (freq) | Chi2 | Fisher's-p | Pearson's-p | Odds Ratio [95%CI] |
| --- | --- | --- | --- | --- | --- | --- |
| **Catabolic pathway** |  |  |  |  |  |  |
| PD |  |  |  |  |  |  |
| C-T-G | 0.507 | 0.500 | 0.077 | 0.7816 | 0.7816 | 1.025 [0.859~1.224] |
| C-T-A | 0.155 | 0.160 | 0.136 | 0.7125 | 0.7125 | 0.958 [0.760~1.206] |
| C-C-G | 0.127 | 0.117 | 0.492 | 0.4831 | 0.4831 | 1.097 [0.847~1.419] |
| T-T-G | 0.109 | 0.118 | 0.519 | 0.4712 | 0.4712 | 0.908 [0.859~1.224] |
| T-C-A | 0.010 | 0.014 |  |  |  |  |
| MSA |  |  |  |  |  |  |
| C-T-G | 0.522 | 0.500 | 1.962 | 0.1614 | 0.1614 | 1.185 [0.934~1.502] |
| C-T-A | 0.157 | 0.160 | 0.006 | 0.9393 | 0.9393 | 1.012 [0.747~1.371] |
| C-C-G | 0.100 | 0.117 | 0.580 | 0.4463 | 0.4463 | 0.870 [0.607~1.246] |
| T-T-G | 0.091 | 0.118 | 2.055 | 0.1718 | 0.1517 | 0.763 [0.527~1.105] |
| T-C-A* | 0.010 | 0.014 |  |  |  |  |
| **Anabolic pathway** |  |  |  |  |  |  |
| PD |  |  |  |  |  |  |
| T-C | 0.420 | 0.416 | 0.067 | 0.7963 | 0.7963 | 1.016[0.902-1.114] |
| T-T | 0.411 | 0.412 | 0.010 | 0.9215 | 0.9214 | 0.994[0.882-1.120] |
| C-C | 0.090 | 0.074 | 3.440 | 0.0637 | 0.0637 | 1.227[0.988 -1.524] |
| C-T | 0.080 | 0.097 | 4.470 | 0.0345 | 0.0345 | 0.802[0.653-0.984] |
| MSA |  |  |  |  |  |  |
| T-C | 0.459 | 0.416 | 4.686 | 0.0305 | 0.0304 | 1.189[1.016-1.391] |
| T-T | 0.366 | 0.412 | 5.2728 | 0.0167 | 0.0167 | 0.822[0.700-0.965] |
| C-C | 0.089 | 0.074 | 1.874 | 0.1711 | 0.1710 | 1.217[0.918-1.613] |
| C-T | 0.087 | 0.097 | 0.876 | 0.3494 | 0.3494 | 0.878[0.669-1.153] |

*haplotypes of catabolic pathway composed by rs1799836-rs1611115-rs4680, and haplotypes of anabolic pathway composed by rs6356-rs921451

**Supplementary Table 3** Genotypic distribution of five polymorphisms in individuals with Parkinson’s disease in the presence or absence of motor fluctuation, dyskinesia, freezing gait and festinating gait

| Variants | Motor Fluctuation | |  | *p* |  | Dyskinesia | | *p* |  | Freezing gait | | *p* |  | Festinating gait | | *p* |
| --- | --- | --- | --- | --- | --- | --- | --- | --- | --- | --- | --- | --- | --- | --- | --- | --- |
|  | Present | Absent |  |  |  | Present | Absent |  |  | Present | Absent |  |  | Present | Absent |  |
| rs1799836* | 19,77 | 106,501 |  | 0.579 |  | 3,22 | 122,556 | 0.615 |  | 54,244 | 71,334 | 0.840 |  | 37,180 | 88,398 | 0.735 |
| rs1799836^#^ | 2,27,53 | 23,182,363 |  | 0.752 |  | 1,20,29 | 24,189,387 | 0.395 |  | 11,81,152 | 15.128.264 | 0.755 |  | 8,49,102 | 17,160,314 | 0.642 |
| rs1611115 | 5,59,123 | 38,355,921 |  | 0.430 |  | 6,25,48 | 47,387,987 | 0.114 |  | 17,255,396 | 26,258,643 | 0.957 |  | 10,110,285 | 33,303,754 | 0.809 |
| rs4680 | 9,72,106 | 74,527,716 |  | 0.799 |  | 4,27,49 | 78,568,767 | 0.468 |  | 29,220,320 | 54,375,500 | 0.619 |  | 26,155,225 | 57,440,595 | 0.569 |
| rs6356 | 5,51,130 | 47,354,914 |  | 0.824 |  | 3,23,52 | 49,377,986 | 0.842 |  | 17,147,404 | 34,256,637 | 0.561 |  | 13,121,271 | 38,282,770 | 0.299 |
| rs921451 | 59,78,51 | 358,626,334 |  | 0.282 |  | 21,34,25 | 393,665,357 | 0.481 |  | 158,262,150 | 258,438,234 | 0.869 |  | 121,183,102 | 241,571,228 | 0.546 |

*in male, ^#^ in female
